# Milk: an epigenetic amplifier of FTO-mediated transcription? Implications for Western diseases

**DOI:** 10.1186/s12967-015-0746-z

**Published:** 2015-12-21

**Authors:** Bodo C. Melnik

**Affiliations:** Department of Dermatology, Environmental Medicine and Health Theory, University of Osnabrück, Sedanstrasse 115, 49090 Osnabrück, Germany

**Keywords:** Cancer, Diabetes, DNMT, Epigenetics, FTO, Milk, miRNA-29, mTORC1, *N*^*6*^-Methyladenosine, Obesity, Transcriptome

## Abstract

Single-nucleotide polymorphisms within intron 1 of the FTO (fat mass and obesity-associated) gene are associated with enhanced FTO expression, increased body weight, obesity and type 2 diabetes mellitus (T2DM). The *N*^*6*^-methyladenosine (m^6^A) demethylase FTO plays a pivotal regulatory role for postnatal growth and energy expenditure. The purpose of this review is to provide translational evidence that links milk signaling with FTO-activated transcription of the milk recipient. FTO-dependent demethylation of m^6^A regulates mRNA splicing required for adipogenesis, increases the stability of mRNAs, and affects microRNA (miRNA) expression and miRNA biosynthesis. FTO senses branched-chain amino acids (BCAAs) and activates the nutrient sensitive kinase mechanistic target of rapamycin complex 1 (mTORC1), which plays a key role in translation. Milk provides abundant BCAAs and glutamine, critical components increasing FTO expression. CpG hypomethylation in the first intron of *FTO* has recently been associated with T2DM. CpG methylation is generally associated with gene silencing. In contrast, CpG demethylation generally increases transcription. DNA *de novo* methylation of CpG sites is facilitated by DNA methyltransferases (DNMT) 3A and 3B, whereas DNA maintenance methylation is controlled by DNMT1. MiRNA-29s target all DNMTs and thus reduce DNA CpG methylation. Cow´s milk provides substantial amounts of exosomal miRNA-29s that reach the systemic circulation and target mRNAs of the milk recipient. Via DNMT suppression, milk exosomal miRNA-29s may reduce the magnitude of *FTO* methylation, thereby epigenetically increasing FTO expression in the milk consumer. High lactation performance with increased milk yield has recently been associated with excessive miRNA-29 expression of dairy cow mammary epithelial cells (DCMECs). Notably, the galactopoietic hormone prolactin upregulates the transcription factor STAT3, which induces miRNA-29 expression. In a retrovirus-like manner milk exosomes may transfer DCMEC-derived miRNA-29s and bovine FTO mRNA to the milk consumer amplifying FTO expression. There is compelling evidence that obesity, T2DM, prostate and breast cancer, and neurodegenerative diseases are all associated with increased FTO expression. Maximization of lactation performance by veterinary medicine with enhanced miRNA-29s and FTO expression associated with increased exosomal miRNA-29 and FTO mRNA transfer to the milk consumer may represent key epigenetic mechanisms promoting FTO/mTORC1-mediated diseases of civilization.

## Background

*FTO*, fat mass- and obesity-associated gene (MIM 612938) maps to chromosome 16q12.2 and is widely expressed in a variety of human tissues with highest levels detected in the brain, pancreatic islets, and the liver [1, 2]. The 505 amino acid-long human FTO and its orthologs are present in vertebrate evolution for at least 450 million years [3]. FTO mRNA is most abundant in hypothalamic nuclei governing energy balance [4, 5]. FTO was identified as an obesity susceptibility gene by several large-scale genome association studies [1, 6, 7]. Single nucleotide polymorphisms (SNPs) in the first intron of FTO are highly associated with obesity and obesity-related traits [1, 6, 7]. In various populations *FTO* has been confirmed to be a major risk gene promoting obesity [8–19]. Obesity is a well-known risk factor for the development of type 2 diabetes mellitus (T2DM). Indeed, FTO has been identified as a critical T2DM susceptibility locus [20–28]. Obesity and T2DM-associated genetic variations of FTO are associated with increased primary transcript levels of FTO mRNA [14, 29, 30].

Not only genetic polymorphisms of FTO, but also the methylation status of FTO, especially CpG hypomethylation of intron 1 has been linked to increased T2DM prevalence [31]. It is not known whether demethylated CpG loci in intron 1 map directly to regulatory regions and SNPs. Notably, FTO methylation in human pancreatic islets of T2DM patients is significantly reduced compared to healthy controls [32]. Thus, not only genetic but also epigenetic modifications of *FTO* appear to modify FTO expression. It is well appreciated that dietary factors induce epigenetic alterations, which have pivotal long-term biological consequences [33].

This paper highlights the potential role of milk as an epigenetic modifier of the human genome paying special attention to cow milk-mediated overactivation of FTO and its impact on the transcriptome of the human milk consumer.

## Review

### FTO regulates fetal and postnatal growth

The FTO gene is widely expressed in both fetal and adult tissues [1]. The mouse mutant *Fused toes* (Ft) is a dominant trait characterized by partial syndactyly of the forelimbs and massive thymic hyperplasia in heterozygotes [34]. Homozygous Ft/Ft embryos die at midgestation and exhibit absent Fto expression in fibroblasts [35]. Fto-null mice exhibit postnatal growth retardation and a significant reduction in adipose tissue and lean body mass [36]. Mice lacking Fto display postnatal growth retardation with shorter body length, lower body weight, lower bone mineral density, and reduced serum levels of insulin-like growth factor 1 (IGF-1) [37]. Remarkably, specific *Fto* deletion in the central nervous system (CNS) results in a similar phenotype as whole body *Fto* deletion pointing to a crucial role of Fto in the CNS to promote postnatal growth [37]. Studies of human cultured skin fibroblasts from subjects with an R316Q mutation that inactivates FTO enzymatic activity showed impaired proliferation and accelerated senescence [2].

Milk is the exclusive nutrient environment provided by mammals promoting postnatal growth during the lactation period [38]. Milk activates the nutrient-sensitive kinase mechanistic target of rapamycin complex 1 (mTORC1), which induces mTORC1-dependent translation [39]. FTO plays a crucial role in mRNA transcription [40], a requirement for mTORC1-dependent translation. Thus, from a mechanistic point of view milk has to interact with both FTO and mTORC1 of the milk recipient.

### FTO controls energy homeostasis and protein intake

In mice, overexpression of Fto leads to a dose-dependent increase in body and fat mass, irrespective of whether mice are fed a standard or a high-fat diet [41]. However, mice with increased Fto expression on a high-fat diet develop glucose intolerance [41]. FTO plays a critical role in controlling feeding behavior and energy expenditure [42]. SNPs of FTO have been linked to higher energy intake and increased appetite [40, 43–49]. FTO mRNA is present mainly in sites related to hunger/satiation control [50]. Changes in hypothalamic FTO expression are associated with cues related to energy intake [50]. Fasting induced cytoplasmic Fto expression in some neurons of rat hypothalamus [51], whereas under conditions of nutrient availability Fto is concentrated in nuclear speckles [30]. Interestingly, FTO has been found to mediate circadian rhythms and inhibits CLOCK-BMAL1-induced transcription [52]. The BMI-increasing allele of FTO showed a significant association with higher dietary protein intake [53].

### Epigenetic regulation of FTO

The FTO and RPGRIP1L genes are located on the long arm of chromosome 16 and share a CpG island with 51 CpG dinucleotides. The obesity-associated SNPs of FTO are located in intron 1 [30]. FTO methylation has been linked to environmental influences such as dietary factors [29]. It is generally accepted that CpG hypomethylation increases transcriptional activity, whereas DNA CpG methylation is associated with gene silencing [54]. Recent evidence indicates that CpG hypomethylation is generally associated with gene silencing at CpG-island promotors of genes, whereas high levels of intragenic methylation are associated with increased transcription [55, 56]. There is compelling evidence that FTO SNPs of intron 1 are associated with increased levels of FTO expression [14, 29, 30]. Toperoff et al. [57] demonstrated that a CpG site in the first intron of the FTO gene showed small but significant hypomethylation in T2DM patients relative to controls. The same group later confirmed that CpG sites in the first intron of FTO of peripheral blood leukocytes exhibited significant hypomethylation in T2DM cases relative to controls [31]. Furthermore, significant FTO hypomethylation has been confirmed in human pancreatic islets of T2DM patients compared to healthy controls [32]. Importantly, decreased methylation of FTO CpG11 sites have been associated with increased FTO mRNA expression [58]. Thus, preliminary indirect evidence supports the view that hypomethylation of specific CpG sites of the *FTO* gene enhance FTO expression. Thus, a critical question arises: Do we have evidence that milk, the exclusive and sufficient nutrient environment of a newborn mammal, shape the epigenome and transcriptome of its recipient by increasing FTO expression?

### Milk miRNA-29s: potential suppressors of DNA methyltransferases

Secreted exosomal miRNAs represent an important layer of gene regulation and intercellular communication [59–64]. MiRNAs bind through partial sequence homology to the 3′-untranslated region (UTR) of their target mRNAs and cause either translational block or mRNA degradation [64]. Melnik et al. [38] have suggested that milk functions like an “epigenetic transfection system” via transfer of milk-derived exosomes. Of all human body fluids, milk contains the highest amount of RNAs [65]. In fact, mRNA- and miRNA-containing exosomes have been discovered in human milk [66–69], bovine milk [70–74], and milk of other mammals [75–78]. An accumulating body of evidence supports the view that milk miRNAs are bioactive compounds in foods [79]. Whereas a recent study in a mouse model screening for selected mouse milk miRNAs (miRNA-375, miRNA-200c/141) revealed rapid milk miRNA degradation in the intestinal fluid [80], Wolf et al. [81] demonstrated that bovine milk exosomes including miRNA-29b and miRNA-200c are taken up by human intestinal cells via endocytosis depending on exosome and cell surface glycoproteins. It has been confirmed in the suckling wallaby that highly expressed milk miRNAs are detected at signifcant higher levels in the neonate blood serum, confirming that milk miRNAs are absorbed in the gut of the young [78]. Milk is obviously a unique mammalian transfer system of exosomal miRNAs and mRNAs from the mother to her infant [38, 82]. MiRNAs of commercial cow´s milk survive processing such as pasteurization, homogenization and refrigeration for at least 2 weeks [83]. Remarkably, bovine milk exosomes are highly resistant against harsh degrading conditions [72, 77]. Bovine milk exosomes containing miRNAs and mRNAs are taken up by human macrophages [72]. Baier et al. [84] provided evidence that cow’s milk miRNAs are absorbed in biologically meaningful amounts from nutritionally relevant doses of cow’s milk and affect gene expression in peripheral blood mononuclear cells, HEK-293 kidney cell cultures, and mouse livers. Importantly, bovine milk miRNA-29b, which is identical with human miRNA-29b (http://www.microrna.org), is taken up by blood mononuclear cells of the milk consumer in a dose-dependent manner and modifies mRNA expression such as RUNX2 [84]. These observations imply that milk-derived miRNA-29b may also affect other miRNA-29b targets such as the mRNAs of the DNA demethylase (DNMT) 3A and 3B [85]. Enforced expression of miRNA-29b results in marked reduction of the expression of DNMT1, DNMT3A, and DNMT3B at both mRNA and protein levels.

It has been demonstrated that miRNA-29b indirectly downregulates DNMT1 by targeting Sp1, a transactivator of *DNMT1* [86–90]. MiRNA-21, another abundant miRNA type of bovine milk [70, 74] which shares sequence homology with human miRNA-21 (http://www.microrna.org), indirectly downregulates DNMT1 by targeting RASGRP1 [91]. It is noteworthy to mention that murine mammary miRNA-21 expression is controlled by prolactin-induced upregulation of STAT5 [92].

Thus, milk appears to modify the infant’s epigenome via suppressing DNA de novo methylation (DNMT3A and DNMT3B) as well as DNA maintenance methylation (DNMT1) [54]. Physiologically, milk consumption is an early life environmental exposure that may modify epigenetic signatures [93]. DNA methylation plays a critical role in genomic imprinting during preimplantation development [94–100] as well as during early differentiation [101]. Remarkably, early nutrition during the postnatal period apparently influences the adult phenotype via DNA methylation [102–104]. It is conceivable that milk via miRNA-29b/miRNA-21/DNMT signaling promotes CpG demethylation at intron 1 of *FTO* resulting in increased expression of FTO, which functions as a critical amplifier of the transcriptional machinery for postnatal growth.

Human breast milk also contains and transfers exosomal miRNA-29b and miRNA-21 to the suckling infant [66–69, 105]. At present, no quantitative studies are available which allow a comparison of the amounts of miRNA-29b and miRNA-21 of commercial cow´s milk and human breast milk, respectively.

### Essential amino acids increase FTO expression

Milk proteins are a rich nutrient source of branched-chain essential amino acids (BCAAs) and glutamine [106, 107]. In mouse hypothalamic N46 cells, mouse embryonic fibroblasts (MEFs) and in human HEK293 cells, FTO mRNA and protein levels are significantly downregulated by total amino acid deprivation [108]. Remarkably, FTO functions as an amino acid sensor [109] and couples BCAA availability to mTORC1 signaling [110], which plays a crucial role in translation [39]. In contrast, FTO knockdown results in the upregulation of genes involved in cellular response to starvation [30] such as *ATG5* and *BECN1*, which initiate autophagy by inhibiting mTORC1 and mTORC1-dependent translation [111, 112].

The availability of BCAAs plays a fundamental role in mTORC1 activation [113–120]. The rate-controlling and irreversible step of BCAA catabolism is catalyzed by the multienzyme mitochondrial branched-chain α-keto acid dehydrogenase (BCKD) [121]. The dihydrolipoyl transacylase (E2) forms the core of the BCKD complex and is of critical importance for BCKD activity [122]. Notably, miRNA-29b targets the mRNA of dihydrolipoyl transacylase (E2) and thereby inhibits BCKD activity and mitochondrial BCAA catabolism [123]. Thus, milk miRNA-29b enhances the availability of BCAAs, an important requirement for FTO expression [108]. It is conceivable that milk-mediated transfer of BCAAs and miRNA-29b-mediated preservation of BCAAs may enhance the recipient´s FTO- and mTORC1 activity.

It is important to note that human breast milk contains 1.2 g/100 mL milk protein in comparison to cow´s milk that contains 3.5 g protein/100 mL. In comparison to human breast milk, equivalent volumes of cow´s milk transfer three times more BCAAs to the milk consumer [38, 39] and thus may overstimulate BCAA-driven FTO activation.

### FTO controls the RNA methylome

FTO belongs to the superfamily of Fe(II) 2-oxoglutarate (2-OG)-dependent dioxygenases [4]. FTO catalyzes the demethylation of 3-methylthymine and 3-methyluracil in single-stranded DNA and RNA to thymine and uracil [124]. Jia et al. [125–127] recently detected that *N*^6^-methyladenosine (m^6^A) in nuclear RNA is a major substrate of the demethylase FTO. The m^6^A mark is the most prevalent internal (non-cap) modification present in mRNAs of all higher eukaryotes [128, 129]. The discovery that FTO is an m^6^A demethylase indicates that this modification is reversible and dynamically regulated, suggesting that reversible RNA methylation may affect gene expression and cell fate decisions by modulating multiple RNA-related cellular pathways, which potentially provide rapid responses to environmental signals such as nutrient and especially milk availability in mammals [129] (Table 1).

**Table 1:** Biological impacts of FTO from fetal to adult life

FTO expression	Biological alterations	References
Increased placental FTO expression	Increased fetal and birth weight	[226–228]
Absent FTO expression (fused toes mutant mice)	Murine embryos die at midgestation	[34, 35]
Fto-null mice	Postnatal growth retardation, reduced adipose tissue and lean body mass, shorter body length, lower bone mineral density, lower serum IGF-1	[36, 37]
Fto deletion in murine CNS	Postnatal growth retardation	[37]
SNPs with higher FTO expression	Higher energy intake, increased appetite, higher dietary protein intake	[40, 43–49, 53]
Hypothalamic FTO expression	Regulation of hunger/satiation, energy intake and circadian rhythm	[50–52]
Early hypothalamic FTO over-expression in the rat	Postweaning hyperphagia	[155]
Fto over-expression in mice	Increase in body and fat mass, glucose intolerance during high-fat diet, metabolic syndrome	[41, 213]
FTO over-expression	Increased gluconeogenesis	[266]
FTO over-expression	Increased adipogenesis, reduced thermogenesis of preadipocytes, insulin resistance	[146, 249, 251, 268]
FTO deficiency in adipocytes	Increased expression of UCP-1 (thermogenin), induction of BAT phenotype with increased thermogenesis	[252]
Obesigenic FTO variants	Increased BMI in children at 8 yrs, higher risk of early menarche at 12 yrs	[234, 244]
Obesigenic FTO variants	Increased BMI and obesity in adults	[8–19]
Obesigenic FTO variants	Increased risk of T2DM in adults	[20–28]
FTO rs9939609 A-allele	Increased risk of coronary heart disease	[214, 215]
Obesigenic FTO variants	Increased risk of cancer, especially PCa and BCa	[276, 277, 291–298]
Obesigenic FTO SNPs	Reduction in frontal lobe volume of the brain, impaired verbal fluency, increased risk of AD	[319–323]
FTO rs9939609 SNP	Reduced leucocyte telomere length, accelerated aging	[342]

Berulava et al. [30] demonstrated that FTO is highly enriched in nuclear speckles, which serve as for storage and modification of pre-mRNA splicing factors. Proposed functions for m^6^A modification include mRNA splicing, export, stability, and immune tolerance [130]. FTO overexpression greatly reduced the levels of m^6^A in cellular RNA underlining that m^6^A is a major physiologic target of FTO [125–127].

Wang et al. [131] recently demonstrated that m^6^A is selectively recognized by the human YTH domain family 2 (YTHDF2) protein to promote mRNA degradation. Liu et al. [132] showed that m^6^A-dependent RNA structural switches regulate RNA–protein interactions. m^6^A alters the local structure in RNA and long non-coding RNA to facilitate binding of heterogeneous nuclear ribonucleoprotein C (HNRNPC), an abundant nuclear RNA-binding protein responsible for pre-mRNA processing [133–137]. These m^6^A-switch-regulated HNRNPC-binding activities affect the abundance as well as alternative splicing of target mRNAs, underlining the pivotal regulatory role of m^6^A-switches on gene expression and RNA maturation [132]. Thus, dynamic m^6^A modifications are recognized by selective binding proteins, which affect the translation status and lifetime of mRNAs. m^6^A methylation not only occurs in mRNA but also in ribosomal RNA (rRNA), transfer RNA (tRNA), small nucleolar RNA (snoRNA), long non-coding RNA (lncRNA), and miRNAs [138–142]. Overexpression of FTO in HeLa cells reduced the level of m^6^A in purified poly (A) RNA by 18 %, whereas *FTO* knockdown increased m^6^A levels by 23 % in poly (A) mRNA [138, 143]. Adenosine methyltransferases (‘writers’), m^6^A demethylating enzymes (‘erasers’) such as FTO and ALKBH5 and m^6^A-binding proteins (‘readers’) thus define cellular pathways for the post-transcriptional regulation of mRNAs. The m^6^A methylation pattern thus constitutes the mRNA ‘epitranscriptome’ [138, 143].

Furthermore, the m^6^A status modifies the interaction of miRNAs with their target mRNAs. A significant portion of m^6^A occurs in close vicinity to the 3´UTRs of mRNA transcripts, which correlate with their miRNA binding sites [138]. Knockdown of FTO affects the steady state levels of several miRNAs pointing to a further layer of FTO-dependent posttranscriptional regulation of gene expression [142]. As miRNAs inhibit their target mRNAs, decreased expression of FTO suppresses global mRNA transcription, whereas overexpression of FTO enhances transcriptional activity. Moreover, m^6^A marks act as a key post-transcriptional modification initiating miRNA biogenesis [144].

Milk, lactation’s nutrient system operating during the postnatal growth period between mother and infant may shape the infants epitranscriptome via RNA m^6^A demethylation to increase transcription, required for postnatal growth, body mass, protein and fat mass accretion.

### FTO regulates adipogenesis-related RUNX1T1 mRNA splicing

Recent evidence links FTO overexpression to enhanced expression of the pro-adipogenic short isoform of the transcription factor RUNX1T1. FTO controls mRNA splicing by regulating the ability of the splicing factor SRSF2 to bind to mRNA in an m^6^A-dependent manner [145]. The pro-adipogenic short isoform of RUNX1T1 stimulates mitotic clonal expansion (MCE) of MEFs and thus enhances adipocyte numbers [146]. In contrast, mRNA m^6^A methylation downregulates adipogenesis in porcine adipocytes [147]. Thus, FTO negatively regulates m^6^A levels and positively regulates adipogenesis, while methyltransferase-like 3 (METTL3), which catalyzes the formation of m^6^A in RNA [148], positively correlates with m^6^A levels and suppresses adipogenesis [147]. In accordance, long-term feeding of commercial pasteurized cow´s milk to young mice increased body weight, caloric intake, and epididymal fat mass as compared to milk-free controls [149].

### FTO controls appetite via ghrelin mRNA demethylation

The “hunger hormone” ghrelin functions as a neuropeptide in the CNS and regulates energy homeostasis [150, 151]. Ghrelin increases appetite by triggering receptors in the arcuate nucleus [152]. The ghrelin receptor is expressed in many brain areas important for feeding control, including hypothalamic nuclei involved in energy balance regulation and reward-linked areas such as the ventral tegmental area (VTA) [153]. It has recently been recognized that ghrelin signaling at the level of the mesolimbic system is one of the key molecular substrates that provides a physiological signal connecting gut and reward pathways [153]. In some way, milk intake should trigger ghrelin signaling enhancing appetite and reward pathways to secure postnatal food intake for mammalian development.

Karra et al. [154] found that subjects homozygous for the FTO “obesity-risk” rs9939609 A allele have dysregulated circulating levels of acyl-ghrelin and attenuated postprandial appetite reduction. FTO overexpression reduced ghrelin mRNA m^6^A methylation, concomitantly increasing ghrelin mRNA and peptide levels. Peripheral blood cells from homozygous (AA) subjects for the FTO “obesity-risk” rs9939609 variant exhibited increased FTO mRNA, reduced ghrelin m^6^A mRNA, and increased ghrelin mRNA abundance [154].

At weaning, hypothalamic FTO mRNA expression was increased significantly in the offspring of obese female Sprague–Dawley rats and FTO was correlated with both visceral and epididymal fat mass [155]. In these rats early hypothalamic FTO overexpression contributes to postweaning hyperphagia [155]. Among carriers of the risk allele of the FTO SNP rs9939609 an association was found between BMI growth and the duration of exclusive breastfeeding (EXBF). In girls, EXBF interacts with the SNP at baseline and can reverse the increase in BMI. In boys, EXBF reduces BMI both in carriers and non-carriers of the risk allele with an association found after 10 years of age. Six months of EXBF will put the boys’ BMI growth curves back to the normal range [156]. Thus, human breast milk appears to control the appropriate postnatal magnitude of FTO expression and activity.

### FTO couples leucyl-tRNA synthase to mTORC1

FTO expression is of pivotal importance for mTORC1 signaling and represents the critical mechanistic link between transcription and translation. MEFs of FTO-deficient mice (*Fto*^−*/*−^ mice) exhibit slower growth rates and reduced mRNA translation compared with wild-type MEFs [110]. Postnatal growth retardation and significant reduction in adipose tissue and lean body mass has been observed in *Fto*^−*/*−^ mice [36]. Severe growth retardation has also been observed in humans with homozygous FTO loss-of-function mutations [2]. During tRNA charging with amino acids amino-acyl-tRNA synthetases (AARS) work together as part of the multi-synthetase complex (MSC), which is essential for mRNA translation [157–159]. In higher eukaryotic systems, several different AARSs including leucyl-tRNA synthetase form a macromolecular protein complex with three nonenzymatic cofactors AIMP1/p43, AIMP2/p38, and AIMP3/p18 [159]. Notably, *Fto*^−*/*−^ MEFs exhibit reduced protein levels of MSC components [110]. A key role for mTORC1 activation plays leucyl-tRNA synthase (LRS) [160, 161]. LRS has been proposed to function as an intracellular amino acid sensor [161]. In yeast, LRS Cdc60 interacts with the Rag GTPase Gtr1 of the EGOC in a leucine-dependent manner [160]. This interaction is necessary and sufficient to mediate leucine signaling to TORC1 and is disrupted by the engagement of Cdc60 in editing mischarged tRNA(Leu) [160]. Han et al. [161] demonstrated that LRS directly binds to Rag GTPase, the mediator of amino acid signaling to mTORC1, in an amino acid-dependent manner and functions as a GTPase-activating protein (GAP) for Rag GTPase to activate mTORC1. Importantly, FTO-deficient MEFs exhibit reduced LRS protein expression [110].

It is tempting to speculate that FTO-mediated demethylation of m^6^A in leucyl-tRNA may modify LRS regulatory functions. Furthermore, Lo et al. [162] detected splice variants of LRS, which differ in catalytic activity. It is possible that LRS mRNA splice variants modify the affinity for leucine binding, thereby change the functional interaction of LRS with Rag GTPase activating mTORC1. This mRNA-splicing dependent mechanism may resemble the generation RUNX1T1 short splice variants observed in the regulation of adipogenesis [145, 146] (Table 2).

**Table 2:** Biological functions of the m^6^A demethylase FTO

Epigenetic FTO functions	Transcriptional effects	References
FTO-catalyzed demethylation of m^6^A in mRNAs	Increased transcription and mRNA stability, modification of m^6^A-dependent alternative splicing and miRNA binding to target mRNAs	[125–127, 138–143]
FTO over-expression	Generation of the pro-adipogenic short isoform of RUNX1T1 promoting MCE increasing adipocyte numbers	[145, 146]
Increased FTO mRNA	Reduction of ghrelin mRNA m^6^A methylation resulting in increased ghrelin mRNA abundance	[154]
FTO-deficient MEFs	Reduced protein expression of the mTORC1 activator leucyl-tRNA synthetase	[110]
Increased FTO expression	Association of *FTO* CpG hypomethylation in T2DM with recuded m^6^A levels in mRNA of T2DM patients	[31, 32, 265]
FTO over-expression	Increased expression of C/EBPβ mRNA, the key transcription factor of gluconeogenesis and adipogenesis	[266, 267]
FTO over-expression	Loss of stem cell self-renewal capability	[207]
FTO over-expression	Increased expression of PRL mRNA enhancing PIP expression involved in PC and BC progression	[185–187]
FTO over-expression	Interaction with APO ϵ4, increasing the risk of AD	[321]

### Dairy cattle FTO mutations increase milk yield

mRNA abundance of FTO in mammary epithelial cells has been shown during lactation of the rabbit [163]. Recently, genetic variations of bovine FTO have been identified that increase milk fat and protein yield in German Holstein dairy cattle [164]. Five SNPs and two haplotype blocks in a 725 kb region covering FTO and the neighboring genes RPGRIP1L, U6ATAC, and 5 S rRNA were associated with milk fat and protein yield [164]. Furthermore, Sorbolini et al. [165] confirmed that FTO belongs to the selection signatures in Piemontese and Marchigiana cattle known to affect productive traits.

Physiologically cattle digest grass, whereas high performance dairy cows are fed with soybean and grain concentrates that provide higher amounts of BCAAs promoting FTO expression and FTO-driven transcription increasing lactation performance. Thus, increased expression of FTO in DCMECs increases milk yield.

### Is bovine FTO mRNA retroconverted into the human genome?

**Fig. 1:**
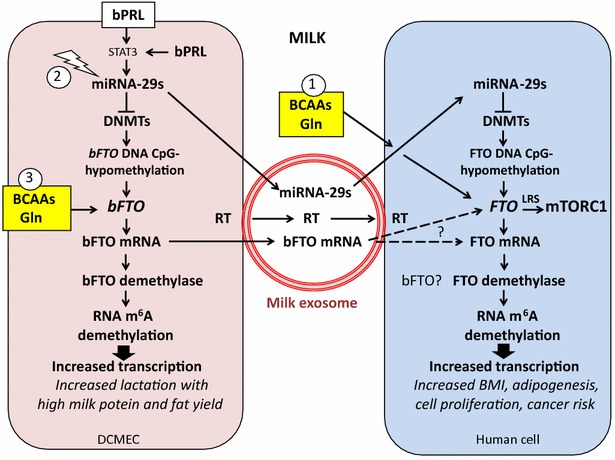
Working model presenting milk-mediated epigenetic activation of FTO-driven transcription. Milk functions in analogy to a retroviral infection via exosome transfer to the cells of the milk recipient. Milk exosomes transport bioactive miRNA-29s that reduce the expression of DNA methyltransferases (DNMTs) resulting in epigenetic activation of human FTO via DNA hypomethylation. Bovine milk exosomes apparently transfer bovine FTO mRNA and reverse transcriptase (RT) that may result in bFTO mRNA retroconversion into the human genome. (*1*) Milk provides abundant amounts of branched-chain amino acids (BCAAs) and glutamine (Gln) that activate FTO and mTORC1. (*2*) Bovine milk production is enhanced by increased expression of miRNA-29s and certain *bFTO* gene polymorphisms. (*3*) Dairy cow feeding with BCAA-enriched concentrated feedingstuffs upregulates bFTO mRNA and protein expression. Efforts of veterinary medicine intended to maximize milk yield associated with increased dairy cow mammary epithelial cell (DCMEC) transcription have an epigenetic impact on FTO expression of the human milk consumer. FTO, the critical m^6^A demethylase, upregulates transcription, adipogenesis, and gluconeogenesis promoting postnatal growth

The human *FTO* gene shares 86.8 % amino acid sequence homology with bovine *FTO* [3]. It is thus conceivable that upregulated bovine FTO mRNA of high performance DCMECs may reach the human milk consumer via intake of mRNA containing milk exosomes. RNA-dependent DNA polymerase activity (reverse transcriptase, RT) has been detected in the milk of several mammalian species including humans [166–173]. Importantly, milk exosomes contain both mRNA and RT [174]. Around 42 % of the human genome is made up of retrotransposons, which operate via RNA intermediates [175]. Recently, Irmak et al. [176] provided translational evidence for the hypothesis that milk exosomes function as an RNA-based gene delivery system between mother and infant. This implies that bovine milk exosomes via transfer of RT and mRNA—such as bovine FTO mRNA—may affect the composition and function of retrotransposons of the human milk consumer. The integration of milk-derived bovine FTO mRNA into the human genome via retrotransposition may be a further mechanism amplifying FTO gene expression [177] (Fig. 1). Most transposable elements are silenced by CpG methylation. Potential milk-miRNA-29/DNMT-mediated changes of the epigenetic state of transposable elements may affect regions encompassing neighboring genes [102]. Evidence has been presented that transposable elements are targets for early nutritional effects on epigenetic gene regulation [102].

### Bovine miRNA-29 increases lactation performance

MiRNA-29a and miRNA-29b have been identified as important miRNAs involved in the regulation of lactation [178, 179]. Bian et al. [180] investigated the roles of miRNA-29s in epigenetic regulation of DCMECs. They showed that miRNA-29s regulate the DNA methylation level by inversely targeting both DNMT3A and DNMT3B in DCMECs. MiRNA-29 s stimulate lactation via decreasing the promoter methylation of *CSN1S1* (casein-α s1), *EIF5* (E74-like factor 5), *PPARγ* (peroxisome proliferator-activated receptor-γ), *SREBP1* (sterol regulatory element binding protein-1) and *GLUT1* (glucose transporter 1) and increase triglyceride biosynthesis [180]. In contrast, inhibition of miRNA-29s causes global DNA hypermethylation and increases the methylation levels of the promoters of important lactation-related genes *CSN1S1*, *ElF5, PPARγ*, *SREBP1* and *GLUT1* and reduces the secretion of lactoprotein, triglycerides and lactose by DCMECs [180]. It is thus conceivable that high expression of bovine miRNA-29b via inhibition of DNMT3A and DNMT3B in high performance dairy cows also decreases bovine *FTO* methylation thereby increasing FTO-dependent transcription. Furthermore, selecting cows with high miRNA-29 expression may increase the miRNA-29 content of commercial milk exosomes. Thus, milk enriched in miRNA-29b and bovine FTO mRNA may epigenetically enhance the FTO expression of the milk consumer (Fig. 1).

### FTO catalyzes prolactin mRNA demethylation

Prolactin (PRL) is the central hormone in female mammals to produce milk [181, 182].

PRL is essential for maintaining lactation and is galactopoietic in dairy cows [183, 184]. m^6^A residues in an intron-specific region of bovine PRL pre-mRNA have been identified [185–187]. Treatment of CHO cells with the methylation inhibitor neplanocin resulted in a 4–6fold increase in nuclear bPRL precursor compared to control cells [186]. FTO-mediated upregulation of bovine PRL expression will enhance the transcription factors STAT3 and STAT5 in DCMECs [188]. Notably, STAT3 promotes the expression of miRNA-29 [189, 190]. In a vicious cycle, miRNA-29-mediated suppression of DNMTs may epigenetically further enhance bovine FTO expression via *FTO* CpG demethylation augmenting lactation. Remarkably, PRL induces the expression of prolactin-inducible protein (PIP), a component of milk that is widely expressed in breast cancer (BC) and prostate cancer (PC) promoting cancer cell growth and proliferation [191–194]. Bovine milk miRNA-29b has been demonstrated to enhance the expression of the osteogenic transcription factor RUNX2 [84], which interacts synergistically with androgen receptor to enhance PIP expression [195]. Thus, FTO-mediated activation of PRL expression may stimulate oncogenic signaling via increased PIP expression.

### Bacterial and viral infections upregulate miRNA-29

Mastitis, the inflammation of mammary glands resulting from bacterial infection, is a common problem in milk production of dairy cattle. Lipopolysaccharide (LPS) injection into murine lactating mammary glands increased the expression of STAT3 [196], promoting miRNA-29 expression [189, 190].

Viral infections such as papilloma virus infections as well enhance miRNA-29 expression via STAT3 signaling [189, 190, 196–198]. It is of most critical concern that oncogenic bovine viruses (e.g. polyoma-, papilloma- or single-stranded DNA viruses) contaminate milk and dairy products [199–201]. Thus, unnoticed infection of dairy cattle may promote epigenetic activation of human FTO via miRNA-29-mediated suppression of DNMTs. Vice versa, overactivated FTO-mediated demethylation of single-stranded DNA and RNA may modify oncogenic viral transcription and mRNA splicing [4, 124, 202, 203]. Furthermore, the transfer of milk exsomes, which resembles a retrovirus-like infection, may promote the spreading of oncogenic bovine viruses.

### FTO-driven diseases of civilization

m^6^A has been identified as a conserved epitranscriptomic modification of eukaryotic mRNAs. Deficiency of m^6^A formation has been proven to affect circadian rhythm, cell meiosis, embryonic stem cell proliferation, and thus is implicated in obesity, cancer and other human diseases [130, 138, 141, 203–213]. FTO is a relevant factor for the development of obesity and the metabolic syndrome in mice [203]. As observed in Swedish men and women, FTO rs9939609 A-allele carriers have an increased risk of coronary heart disease (CHD) [214, 215]. There is evidence in humans that milk consumption correlates with the risk of CHD [216, 217]. It is thus conceivable that increased FTO expression from fetal to adult life promotes diseases of civilization.

#### Fetal macrosomia and increased birth weight

Fetal overgrowth and increased birth weight are risk factors for the development of diseases of civilization such as obesity [218–226]. Sébert et al. [227] identified FTO as a critical factor for fetal programming of obesity-related disorders. The authors found a significant relation between placental FTO gene expression and fetal weight at 110 days gestation [227]. In humans, FTO is highly expressed in the placenta and is associated with increased fetal weight and length [228]. Bassols et al. [228] suggested that FTO controls important genes related to fetal growth. Liu et al. [58] investigated placental FTO expression in relation to the promoter methylation of *FTO* in a Chinese population. Intriguingly, the methylation rates of CpG11 sites were significantly decreased in high birth weight newborns [58]. The investigators concluded that high placental FTO expression is associated with increased birth weight [58]. Milk exosome-mediated transfer of bovine miRNA-29 and miRNA-21 may via downregulation of DNMTs reduce the methylation of critical CpG sites of the FTO promoter explaining milk’s function as an epigenetic enhancer of FTO expression. The Generation R study observed the association of increased fetal and infant growth with an increased risk of obesity during early childhood [229]. A higher peak weight velocity, which generally occurs in the first month after birth, was associated with an increased risk of overweight and obesity at 4 years of age [229]. Accumulating evidence underlines that milk consumption increases placental, fetal and birth weight [230–232].

#### Increased growth trajectories in childhood

The A allele of the FTO rs9939609 SNP is associated with a high BMI from 5.5 years onwards [233]. Established obesity loci including FTO affect the level and the rate of change in BMI at 8 years in children [234]. The National Health and Nutrition Examination Survey (NHANES) provided evidence that milk consumption in children increases BMI [235]. Among children of 5–10 years of age, those in the highest quartile for milk intake had higher BMI [236]. Notably, milk had more consistent positive associations with BMI than any other dairy product, and these are strongest among children of 2–4 years of age [236].

#### Increased linear growth

FTO is highly expressed in the hypothalamus and pituitary gland [1, 2, 236]. FTO controls the somatotropic (growth hormone-IGF-1) axis [37, 236]. *Fto*-deleted mice exhibit reduced serum levels of IGF-1 [37]. Adult obesity susceptibility variants including FTO conferred a faster tempo of height growth that was evident before puberty [237]. Remarkably, milk consumption is known to shift the prepubertal somatotropic axis [238] and increases growth hormone (GH) and total serum IGF-1 levels [239, 240]. It is widely accepted that cow´s milk consumption accelerates linear growth in children [241]. Thus, milk consumption may increase the somatotropic axis in an FTO-dependent manner.

#### Early menarche and diabetes risk

By using a BMI-increasing-allele-score including FTO it has been demonstrated that each 1 kg/m^2^ increase in childhood BMI was predicted to result in a 6.5 % higher absolute risk of early menarche before age 12 years [242]. These findings support a causal effect of BMI on early menarche [242]. It is of considerable concern that early menarche is associated with an increased risk of T2DM and obesity [243, 244]. In fact, NHANES [245] provided evidence that higher milk intake of children is associated with an increased risk of early menarche. However, milk consumption after the age of 9 years did not predict the age at menarche [246], which points to a sensitive window affecting BMI-menarche interactions during early childhood.

#### Obesity

There is compelling evidence that *FTO* is one of the world´s major risk genes increasing BMI and promoting obesity [8–19]. Notably, carriers of the FTO risk allele rs8050136 have an increased risk of CHD mediated by BMI [247]. FTO deficiency in mice led to reduction in adipose tissue [213]. The association between *FTO* and fat mass in humans develops by the postnatal age of 2 years [248]. Striking evidence underlines that FTO-mediated mRNA demethylation regulates mRNA alternative splicing in the control of mitotic clonal expansion (MCE) resulting in adipogenesis [146, 249]. Zhang et al. [250] confirmed that the demethylase activity of FTO is required for 3T3 L1 preadipocyte differentiation. The level of m^6^A is decreased in cells overexpressing FTO [250].

Furthermore, the obesity-associated *FTO* allele represses mitochondrial thermogenesis in adipocyte precursor cells in a tissue-autonomous manner associated with a shift of energy-dissipating beige adipocytes to energy-storing white adipocytes [251]. FTO-deficient adipocytes exhibit a reduced de novo lipogenesis and fourfold higher expression of uncoupling protein 1 (UCP1, thermogenin) mRNA and protein compared with control cells. The upregulation of UCP1 in FTO-deficient adipocytes enhances mitochondrial uncoupling of the respiratory chain, allowing for fast substrate oxidation with a low rate of ATP production [252]. Thus, FTO is not only involved in adipocyte differentiation but also inhibits browning, the development of brown adipose tissue (BAT). BAT was believed to show rapid involution in early childhood, leaving only vestigial amounts in adults. However, recent evidence suggests that its expression in adults is far more common than previously appreciated [253]. Rockstroh et al. [254] detected BAT-like and UCP1-positive adipocytes in 10.3 % of 87 lean children (aged 0.3–10.7 years) and in one overweight infant, whereas they did not find brown adipocytes in obese children or adults. It is conceivable, that during the period of lactation milk-derived FTO signaling reduces UCP1 expression in BAT and thus reduces BAT-driven thermogenesis, a requirement for early postnatal survival. Milk (FTO)-mediated suppression of UCP1 would allow the generation of higher levels of ATP required for biosynthetic pathways during postnatal growth.

Milk provides abundant amounts of BCCAs and glutamine required for FTO expression [108]. According to a recent epidemiological study with 177,330 individuals a positive association between the BMI-increasing allele of FTO variant and higher dietary protein intake has been observed supporting the link between FTO and adiposity and dietary protein intake [53].

Milk via transfer of abundant BCAAs and exosomal miRNA-29 may thus activate FTO expression promoting adipogenic transcription (Fig. 1). In fact, feeding commercial pasteurized cow’s milk increased BMI and fat mass in mice [149]. There is further evidence that milk consumption in children, adolescents and adults increases BMI and obesity [235, 255–258].

#### Type 2 diabetes mellitus

FTO has been confirmed as an important diabetes susceptibility locus [20–28] associated with increased primary transcript levels of FTO mRNA [14, 30]. Patients with the FTO risk allele showed significantly higher serum insulin concentrations and HOMA-IR compared with subjects without the risk allele [259]. Bell et al. [260] were the first who identified variant-CpG restricted haplotype-specific methylation within the FTO T2DM and obesity susceptibility locus tagged by SNP rs8050136. Several studies identified increased expression of the miRNA-29 family as a biomarker for T1DM and T2DM [261–264]. MiRNA-29s via DNMT suppression may decrease the CpG methylation level of FTO in pancreatic β-cells. In fact, recent evidence underlines that increased FTO expression is related to CpG hypomethylation of *FTO* [31, 32]. Toperoff et al. [31] found that DNA methylation of a specified regulatory site in peripheral blood leukocytes (PBLs) is associated with impaired glucose metabolism and T2DM. Dayeh et al. [32] identified 1649 CpG sites and 853 genes, including FTO with differential DNA methylation in T2DM islets. FTO methylation in human pancreatic β-cells of non-diabetic patients differed significantly from FTO methylation of T2DM patients, respectively [32]. Modest differences in DNA methylation of individual CpG sites may exert big effects on gene expression over long periods of time [31, 32]. As DNA hypomethylation commonly increases gene activity [54], it is conceivable that CpG hypomethylation of FTO in T2DM pancreatic β-cells may decrease m^6^A methylation of mRNAs. Indeed, Shen et al. [265] demonstrated that the m^6^A contents in the RNA from T2DM patients and diabetic rats were significantly lower compared with controls. The mRNA expression level of FTO was significantly higher in T2DM patients than that of the controls and was associated with the risk of T2DM. Moreover, the m^6^A contents were inversely correlated with FTO mRNA expression [265].

There is recent evidence that FTO overexpression is also involved gluconeogenesis associated with upregulation of mRNA expression of the rate-limiting gluconeogenic enzymes glucose-6-phosphatase (G6P) and mitochondrial phosphoenolpyruvate carboxykinase (PEPCK) [266]. FTO overexpression resulted in increased mRNA and protein expression of CCAAT/enhancer-binding protein-β (C/EBPβ), which promotes G6P and PEPCK expression [266]. Notably, C/EBPβ activates expression of C/EBPα and PPAR-γ, which further drives the adipocyte phenotype [267] after FTO-driven mitotic clonal expansion (MCE) of adipocytes [146, 249, 250]. Thus, FTO counteracts the activity of metformin, which inhibits gluconeogenesis and adipogenesis, whereas FTO overexpression upregulates gluconeogenesis and adipogenesis.

Interestingly, FTO mRNA levels were increased in subcutaneous adipose tissue (SAT) of T2DM patients, and treatment with Rosiglitazone improved insulin sensitivity and reduced SAT FTO mRNA levels [268]. SAT FTO mRNA and protein levels were increased in insulin resistant women (high HOMA) compared to insulin sensitive women (low HOMA) [268]. Furthermore, FTO expression was transiently increased in early 3T3-L1 adipocyte differentiation, which coincides with the induction of PPARγ_2_ expression [268]. There is compelling evidence that omental adipose tissue (OAT) FTO expression is associated with adiposity, whereas SAT FTO expression is associated with insulin resistance [268].

Remarkably, the EPIC-InterAct Study (n = 340,234) provided evidence for an increased T2DM risk in association with milk consumption in contrast to other dairy products [269]. Obviously, it is a fundamental function of milk to increase FTO-dependent β-cell mRNA transcription and insulin production, which drive mTORC1-dependent translation [39]. However, persistent milk-mediated activation of β-cell transcription and translation will induce premature aging and β-cell endoplasmic reticulum (ER) stress promoting early onset of T2DM [264]. Notably, high intakes of milk, but not meat, increase serum insulin levels and insulin resistance in 8-year-old boys [270]. Early insulin resistance is commonly treated with the anti-diabetic drug metformin, which is a multifunctional inhibitor of mTORC1 [271]. Remarkably, metformin has recently been shown to induce DNA methylation [272], thus may directly counteract milk-mediated epigenetic upregulation of FTO expression.

#### Cancer

FTO polymorphisms can regulate the expression of genes at large kilobases of distance as well as the expression of *FTO* itself, and regions for transcription factor binding. To date it has been observed that variants rs9939609, rs17817449, rs8050136, rs1477196, rs6499640, rs16953002, rs11075995 and rs1121980 are associated with the risk of developing cancer [273].

There is recent interest in the role of m^6^A mRNA methylation in stem cell and cancer stem cell homeostasis [204–207, 212]. m^6^A RNA methylation is required to maintain mouse embryonic stem cells in their ground state [207]. Loss of m^6^A methylation is associated with a loss of self-renewal capability [207]. m^6^A RNA methylation is regulated by miRNAs and promotes reprogramming to pluripotency [209].

Long interspersed element 1 (L1) retrotransposon mobilization occurs exclusively in cancers of epithelial origin [274]. According to a recent hypothesis L1 promoter hypomethylation is correlated with epithelial to mesenchymal transition (EMT) promoting metastasis. Furthermore, human L1 retrotransposition, which is dependent on RNA intermediates, is associated with genetic instability [275]. Milk-derived miRNA-29s via DNMT suppression may be involved in L1 hypomethylation and L1 activation promoting cancer initiation and progression.

#### Prostate cancer

Prostate cancer (PC) is the most common cancer of men in Western societies. The rs9939609 A allele, which was associated with higher BMI, was positively associated with high-grade PC [276]. Machiela et al. [277] identified 10 T2DM markers including FTO that were associated with increased risk for PC. This points to a common genetic or epigenetic basis of T2DM and PC. In fact, an association of T2DM and PC has recently been confirmed in American Indians [278]. During a mean follow-up of 8.5 years, 2446 men of 129,502 participants of the EPIC study developed PC. Waist circumference and waist-hip ratio were positively associated with risk of advanced disease [279].

There is compelling evidence that total dairy protein intake is related to PC risk [280]. According to the EPIC (n = 142,251) daily intake of 35 g dairy protein increased PC risk by 32 % [280]. The Physicians’ Health study provided evidence that only whole milk was consistently associated with higher incidence of fatal PC in the entire cohort and higher PC-specific mortality among cases [281]. In fact, feeding reconstituted milk protein powder but not whole milk in two mouse models of benign and neoplastic lesions did not promote PC progression [282]. Notably, daily milk consumption in adolescence (vs. less than daily), but not in midlife or currently, was associated with a 3.2-fold risk of advanced PC in the population-based Iceland cohort of 8894 men [283]. Thus, there is good reason to suggest that milk-mediated upregulation of FTO increases transcriptional activity driving an mRNA landscape for the development and progression of PC during childhood and adolescence. Moreover, milk-mediated epigenetic activation of FTO may be linked to FTO-driven activation of mTORC1 [110], which is closely involved in the pathogenesis of PC [284, 285]. Indeed, cow’s milk stimulated the growth of LNCaP cells producing an average increase in cancer cell growth rate of over 30 % [286]. Furthermore, FTO-mediated upregulation of PRL may enhance oncogenic PIP expression [192, 193]. There is accumulating evidence for the beneficial effect of metformin in the treatment and prevention of PC, especially in patients with T2DM [287–290]. Metformin´s anti-cancer effects may be traced back to its capability to methylate DNA [272], which may attenuate FTO-PRL-PIP well as FTO-mTORC1 signaling.

#### Breast cancer

Breast cancer (BC) is the most common cancer of women in Western societies and is often associated with obesity [291]. A recent epigenome-wide association study reveals decreased average methylation levels years before BC diagnosis [292].

Genome-wide SNP studies strongly suggest that the *FTO* locus is associated with estrogen receptor (ER)-negative BC [293]. This signal is tagged by rs11075995, located in a 40 kb LD block in intron 1 of *FTO*, within an enhancer region that appears to be active in both normal and triple-negative BC cells. da Cunha et al. [294] observed a 4.59-fold increased risk for women who have the allele combination FTO rs1121980/FTO rs9939609/MC4R rs17782313 indicating an interaction between FTO and MC4R polymorphisms in BC development. FTO rs16953002 AA genotype conferred significant increased BC risk compared to GG genotype in a Chinese population [295]. In another Chinese study 5 susceptibility loci including FTO correlated with BC [296]. Of 41 recently discovered BC susceptibility variants, associations were found between rs1432679 (EBF1), rs17817449 (MIR1972-2: FTO), rs12710696 (2p24.1), and rs3757318 (ESR1) and adjusted absolute and percent mammographic dense areas, respectively [297]. Singh et al. presented a BC cell model featuring an embryo-like gene expression with amplification of FTO [298]. Thus, substantial evidence links FTO to pathogenesis of BC or subtypes of BC.

From 1916 to 1975, BC risk increased 2.7-times in Norway and has been associated with changes of life style factors after World War II including milk intake [299]. In fact, women consuming 0.75 L or more of full-fat milk daily had a relative risk of 2.91 compared with those who consumed 0.15 L or less [300]. Remarkably, consumption of commercial whole and non-fat milk increased the incidence of 7,12-dimethylbenz(a)anthracene (DMBA)-induced mammary tumors in rats [301]. Tumor numbers, volume and incidence doubled after 20 weeks of milk consumption in these DMBA-induced mammary tumors [301]. Notably, PRL binding to DMBA-induced mammary tumors was three times higher than that observed in lactating mammary glands of the rat [302, 303]. Further administration of PRL enhanced tumor growth [302, 303], whereas pharmacological suppression of PRL secretion inhibited DMBA-induced mammary carcinogenesis in the rat [304]. It is of critical concern that large prospective epidemiological studies show correlations between circulating levels of PRL with an increased risk of ER-positive invasive BC [305].

Breastfeeding apparently meets the appropriate species-specific signaling axis of FTO- and mTORC1-mediated postnatal programming. Women, who had been breast-fed have a reduced lifetime risk of developing BC [306]. Breastfeeding reduces the risk of developing BC [307], specifically of triple-negative BC [308].

A large Swedish cohort study demonstrated that people with lactose intolerance and low consumption of milk and other dairy products had a decreased risk of BC, lung, and ovarian cancers [309]. These data imply that cow´s milk consumption increases transcription, translation and cell proliferation in BC. Notably, there is preliminary evidence that metformin treatment reduces BC recurrence in T2DM patients [310, 311]. Metformin-induced DNA methylation [272] may reduce FTO-PRL signaling that increases PIP expression in BC [192, 193]. Remarkably, PIP regulates proliferation of luminal A type BC cells in an ER-independent manner [312], supporting the role of FTO in ER-negative BC [293].

#### Neurodegenerative diseases

Environmental factors such as diet contribute significantly to risk of Alzheimer’s disease (AD) and Parkinson’s disease (PD) [313]. Accumulating evidence points to the important role of dietary epigenetic regulation in the pathophysiology of AD [314–316]. AD and PD are both tauopathies. mTORC1 induces abnormally hyperphosphorylated tau proteins, which aggregate resulting in compromised microtubule stability [317]. mTORC1 is involved in regulating tau distribution in subcellular organelles and in the initiation of tau secretion from cells to extracellular space [318]. Notably, FTO via LRS plays a crucial role for mTORC1 activation [110].

Carriers of common *FTO* polymorphisms rs9939609 A allele exhibit a reduction in frontal lobe volume of the brain and an impaired verbal fluency performance [319, 320]. A population-based study from Sweden found that carriers of the FTO rs9939609 A allele have an increased risk for incident AD [320]. Furthermore, an interaction between FTO and APOE was found, with increased risk for dementia for those carrying both FTO AA and APOE ϵ4 [321]. Genetic variation in introns 1 and 2 of the FTO gene may contribute to AD risk [322]. Remarkably, impaired satiation and increased feeding behavior has been reported in the triple-transgenic AD mouse model [323], which may point to increased FTO expression.

A dietary pattern associated with a lower AD risk was characterized by higher intakes of salads, nuts, fish, tomatoes, poultry, cruciferous vegetables, fruits, and dark and green leafy vegetables and a lower intake of high-fat dairy products, red meat, organ meat, and butter [324].

Overweight is more prevalent in PD patients [325, 326]. In early PD, weight gain was revealed over 3 years accompanied by an increase in fat mass and waist circumference [327]. Whereas FTO gene polymorphisms have not yet been studied in PD patients, several studies show an association between milk consumption and the risk of PD [328–333]. DNA methylation plays a pivotal role in the pathogenesis of age-related neurodegeneration and cognitive defects [334]. Milk-miRNA-29-mediated suppression of DNA methylation and FTO-enhanced mRNA m^6^A demethylation via persistent milk consumption may thus play an important role in the pathogenesis of neurodegenerative diseases.

### Future prospects

The continuous interaction between the individual´s genetic makeup and environmental factors such as early nutrition result in a spectrum of states ranging from healthy aging to age-related diseases of civilization [102–104, 335]. Milk is a highly specialized nutrient and signaling system of mammalian evolution that apparently shapes the epitranscriptome of the milk recipient via FTO-mediated modifications of RNA nucleotides. Future research should unravel milk´s biological impact on the recently recognized dual axis of coordinated regulation between the genome and the epigenome and the transcriptome and the epitranscriptome, respectively [336]. More scientific insights into milk’s epigenetic signature, which links the maternal lactation genome to the infant’s epitranscriptome as well as the impact of pasteurized cow’s milk on the epitranscriptome of the milk consumer will allow a deeper understanding of milk-mediated postnatal programming as well as the pathogenesis of Western diseases of civilization.

## Conclusions

**Fig. 2:**
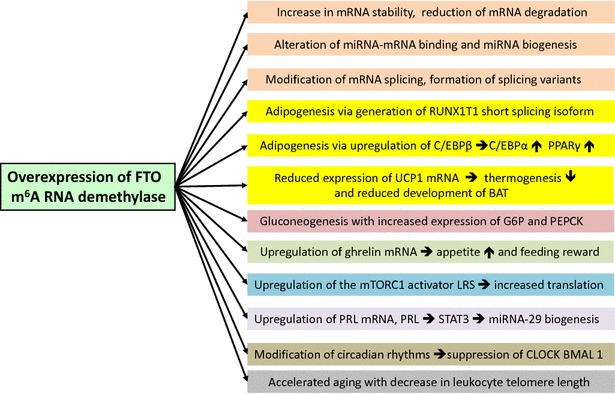
Synopsis of m^6^A mRNA modifications induced by increased FTO expression. The RNA m^6^A eraser FTO drives crucial epigenetic mechanisms fundamentally involved in the pathogenesis of diseases of civilization

Early-life experiences play a critical role for lifelong metabolic programming. Current research focuses on the role of epigenetic causes of excess adult weight gain and early onset of age-related diseases [337]. Importantly, the *FTO* gene has been recognized to play a crucial role in the early-life determination of body weight, body composition and energy balance [337]. Milk is the early nutritional environment and life experience of all mammals. There is increasing evidence that milk is not “just food” but represents a sophisticated signaling system of mammals to promote anabolism for postnatal mTORC1-mediated growth [38]. It has recently been demonstrated that milk stimulates mTORC1-dependent translation [39], a pivotal requirement for cell growth and proliferation. There is overwhelming evidence that the well-preserved gene *FTO* plays a predominant role in DNA demethylation and m^6^A-dependent mRNA demethylation [1, 4, 125, 129–132]. Thus, FTO promotes transcription and increases genomic transcriptional activity, a requirement for postnatal growth. Milk functions apparently as an “epigenetic interface” generated by the lactation genome that controls the FTO-dependent transcriptome of the milk recipient (Fig. 1). FTO is thus an evolutionary checkpoint coordinating the program of the lactation driving transcription, translation and anabolism of the newborn mammal. FTO-mediated demethylation of mRNAs increases transcriptional activity and generates mRNA splice variants that are critically involved in adipogenesis [144–146], ghrelin-regulated appetite control [150], and LRS-mediated mTORC1 activation [110, 157–162] (Fig. 2).

Remarkably, FTO plays a critical role in milk production. The mRNA of PRL, the most important hormone promoting lactation, is regulated via m^6^A methylation [180, 186] and thus depends on FTO activity. In this context it is not surprising that bovine *FTO* variants enhance lactation performance [164]. Moreover, enhanced expression of miRNA-29 in DCMECs increases milk yield [180]. Abundance of miRNA-29s in DCMECs of high performance dairy cows may downregulate DNMT-mediated methylation of bovine *FTO*, thereby increasing bovine FTO mRNA and protein levels of DCMECs. Bovine miRNA-29s, which are identical with human miRNA-29s, and bovine FTO mRNA, which is highly homologous to human FTO mRNA, may reach the consumer of pasteurized fresh milk via uptake of milk exosomes [84]. Viral and bacterial infections of dairy cows may further increase miRNA-29 levels [189, 190, 196–201] (Fig. 1). Thus, the efforts of veterinary medicine intensifying lactation performance and milk yield apparently overstimulate FTO signaling of the human milk consumer, an overlooked interaction in the pathogenesis of Western diseases. The presented working model allows a new understanding of milk as mammal´s temporal amplifier of the epitranscriptome of the milk recipient. m^6^A is a common modification of mRNA with potential roles in fine-tuning the RNA life cycle and mRNA expression control [143, 338]. Milk-activated FTO operates as a methylation “eraser” to promote transcription and translation for adequate growth during the lactation period [129, 143]. It is conceivable that individuals carrying obesigenic genetic *FTO* variants with increased FTO expression may be even more susceptible to milk-mediated epigenetic activation of FTO. FTO should be regarded as the driver of the transcriptome, whereas mTORC1 drives the translational machinery interconnected via an FTO-mTORC1 crosstalk.

Persistent milk-mediated epigenetic FTO signaling may explain the epidemic of age-related diseases of civilization. It is thus not surprising that an increased mortality in relation to high milk intake has recently been observed in a Swedish cohort of men and women [339]. The antagonistic pleiotropy theory of aging postulates that genes beneficial early in life operate at the cost of aging when persistently activated later in life [340]. Persistent overactivation of evolutionary developmental genes, such as *FTO* and *MTOR*, which are most important for perinatal programming, appear to be the major health hazard promoting aging and early onset of age-related diseases. In fact, obesity-related risk allele carriers of *FTO* gene show dose-dependent increments in BMI during aging. Moreover, the obesity-related risk allele is associated with reduced medial prefrontal cortical function during aging [341]. In addition, presence of the FTO rs9939609 polymorphism risk allele in a Korean population was inversely associated with leukocyte telomere length [342].

Future research should characterize the epigenetic FTO-activating potential of milk versus other fermented dairy products. The relative contribution of milk-derived regulatory mechanisms that activate FTO expression such as BCAAs and exosomal miRNAs, have to be determined in detail. It is of critical interest to provide experimental evidence showing that milk consumption modifies the *FTO* methylation status resulting in increased FTO expression and activity. Furthermore, it is important to study differences in epigenetic FTO activation levels between human breast milk, bovine milk, and commercial milk of high performance dairy cows. Special attention should be paid to the effects of bovine milk exosomal miRNAs and mRNAs in the epigenetic control of FTO expression during sensitive periods of pre- and postnatal FTO-mediated metabolic programming.

## Abbreviations

AD: Alzheimer’s disease
AARS: amino-acyl-tRNA synthetase
APOE: apolipoprotein E
ATG5: autophagy 5, S. cerevisiae, homolog of
BAT: brown adipose tissue
BC: breast cancer
BCAA: branched-chain amino acid
BCGN1: beclin 1
BMAL1: brain and muscle ARNT-like protein 1
BMI: body mass index
C/EBPβ: CCAAT/enhancer-binding protein-β
CHD: coronary heart disease
CLOCK: circadian locomotor output cycles kaput
CNS: central nervous system
CpG: cytosine-phosphate-guanine
*CSN1S1*: casein alpha s1 gene
DCMEC: dairy cow mammary epithelial cell
DMBA: 7,12-dimethylbenz(a)anthracene
DNMT: DNA methyltransferase
*EIF5*: E74-like factor 5 gene
EMT: epithelial to mesenchymal transition
EPIC: European Prospective Investigation into Cancer and Nutrition
ER: estrogen receptor
EXBF: exclusive breast feeding
*FTO*: fat mass- and obesity-associated gene
*GLUT1*: glucose transporter 1 gene
HNRNPC: heterogeneous nuclear ribonucleoprotein C
HOMA: homeostasis model assessment
IGF-1: insulin-like growth factor 1
LPS: lipopolysaccaride
LRS: leucyl-tRNA synthase
MCE: mitotic clonal expansion
MEF: mouse embryonic fibroblast
m^6^A: *N*^6^-methyl-adenosine
METTL3: methyltransferase-like 3
miRNA: microRNA
MSC: multi-synthetase complex
mTORC1: mechanistic target of rapamycin complex 1
NHANES: National Health and Nutrition Examination Survey
PD: Parkinson´s disease
PC: Prostate cancer
PIP: prolactin-inducible protein
PRL: prolactin
PPARγ: peroxisome proliferator-activated receptor-γ
rRNA: ribosomal RNA
RT: reverse transcriptase
RUNX2: runt-related transcription factor 2
SAT: subcutaneous adipose tissue
SNP: single nucleotide polymorphism
snRNA: small nuclear RNA
snoRNA: small nucleolar RNA
STAT: signal transducer and activator of transcription
SREBP1: sterol regulatory element binding protein-1
tRNA: transfer RNA
T2DM: type 2 diabetes mellitus
UCP-1: uncoupling protein 1
UTR: untranslated region
VTA: ventral tegmental area
